# Wake-Up Receiver with Equal-Gain Antenna Diversity †

**DOI:** 10.3390/s17091961

**Published:** 2017-08-25

**Authors:** Timo Kumberg, Robert Tannhaeuser, Leonhard M. Reindl

**Affiliations:** Laboratory for Electrical Instrumentation, Department of Microsystems Engineering–IMTEK, University of Freiburg, Georges-Koehler-Allee 106, 79110 Freiburg, Germany; robert.tannhaeuser@imtek.uni-freiburg.de (R.T.); reindl@imtek.de (L.R.)

**Keywords:** wake-up receiver, wireless sensor node, small-scale fading, antenna diversity, wake-up packet, wireless sensor network

## Abstract

Small scale fading signals resulting from multipath propagation can cause signal strength variations in the range of several dB. Resulting from the fluctuating signal strengths, the wake-up packet reception rate can decrease significantly. Using antenna diversity can greatly mitigate these effects. This article presents a novel wireless sensor node with wake-up receiver that uses an equal-gain diversity method with two antennas in the wake-up path. Summation of the two diversity branch signals is done after the passive demodulation of the incoming signals. As a result, the wireless sensor node requires almost no additional active parts that would increase power consumption. Furthermore, we demonstrate experimentally the improved wake-up robustness and reliability achieved by this diversity technique in a multipath environment.

## 1. Introduction

Wireless sensor networks are used in various applications like environmental monitoring, child education, smart manufacturing, infrastructure monitoring and many others. A wireless sensor network usually consists of many small self-powered sensor nodes that measure their environment and communicate data to other nodes or a base station [1].

Recently, more and more wireless sensor networks consist of wireless sensor nodes that include wake-up receivers [2,3,4,5] that reside in a low-power stand-by state until they receive a wake-up massage. Only after receiving this message, or a sensor event, a sensor node wakes up to full operation. This approach can be more favorable, with respect to energy consumption, than duty-cycling [6].

The low-frequency wake-up message is usually modulated on a high-frequency carrier signal using on-off-keying modulation. To ensure ultra-low power consumption, the wake-up message can be demodulated passively [6,7]. For instance, the wireless sensor nodes presented by [6,8] use the AS3932 wake-up receiver that listens permanently to a 125 kHz signal that is modulated on a 868 MHz carrier frequency and demodulated using a passive rectifier and a low-pass filter. Changing between communication and low-power wake-up listening is implemented by using an antenna switch.

However, resulting from the passive demodulation, wake-up receivers usually exhibit a lower sensitivity than communication radios [6,9,10]. To improve robustness and reliability of wake-up messages, Kumberg et al. presented in [11] a technique to transmit concurrent wake-up messages by purposefully interfering signals of two or more wireless sensor nodes. This creates a wake-up signal by using the beat frequency of the superposed and slightly out of tune carrier signals and increases the transmitted signal strength.

In this article, we present a wireless sensor node with wake-up receiver, based on the work presented in [12]. The novel design uses equal-gain diversity in the wake-up path as introduced in Section 2. Superposition of the diversity signals is realized at 125 kHz after the passive demodulation of the incoming signals. As a result, there are almost no additional active parts required to achieve constructive interference as phase-alignment of the signals is not required, as demonstrated in Section 3. In Section 4, we demonstrate experimentally the improved wake-up signal robustness and reliability in multipath propagation environments. Finally, the article is concluded in Section 5.

## 2. Background

Deep and rapid amplitude fluctuations caused by the close surroundings of a receiver are called small-scale fading. In wireless propagation, a signal from an antenna reaches another antenna over several paths with their associated path lengths and attenuations. As a result, many copies of one signal reach the antenna after different delays, where they superimpose each other destructively or constructively [13].

If the bandwidth $B_{s}$ of a transmitted signal is smaller than the bandwidth $B_{C}$ over which a wireless channel has a constant gain and a linear phase response, the transmitted signal will experience flat fading [14]. A flat fading signal is a locally coherent signal. The symbol amplitudes of a transmission will change over time due to flat fading, as much as 30 dB [14]. On the receiver side, an automatic gain control (AGC) can be used to mitigate flat fading effects [15], but, more so, diversity techniques help to mitigate small-scale fading effects [16]. Assuming sufficiently separated signals in time, frequency, space, or polarization, they are independent of each other [17]. Hence, if one antenna does not receive a signal along one branch due to small-scale fading, the other uncorrelated antenna will likely still receive the signal along a diversity branch [16].

According to [17], a diversity system is a system that provides two or more similar copies of the same signal. For example, a receiver detects signal $f_{1}\left(t\right)$ and stores it locally. Then, the receiver detects signal $f_{2}\left(t\right)$. By combining $f_{1}\left(t\right)$ and $f_{2}\left(t\right)$, that is $f\left(t\right)=f_{1}\left(t\right)+f_{2}\left(t\right)$, the signal quality can be improved.

Resulting from additional noise present in the wireless channel, signal $f_{1}\left(t\right)$ is a combination of the desired signal $s_{1}\left(t\right)$ and a noise signal $n_{1}\left(t\right)$, that is $f_{1}\left(t\right)=s_{1}\left(t\right)+n_{1}\left(t\right)$, and likewise is true for signal $f_{2}\left(t\right)$. Therefore, $f\left(t\right)=s_{1}\left(t\right)+s_{2}\left(t\right)+n_{1}\left(t\right)+n_{2}\left(t\right)$ is a combination of the desired signal and the present noise. In a diversity system, $s_{1}\left(t\right)$ and $s_{2}\left(t\right)$ are similar to each other and combining $s_{1}\left(t\right)$ and $s_{2}\left(t\right)$ increases the desired signal amplitude. Assuming $n_{1}\left(t\right)$ and $n_{2}\left(t\right)$ to be independent additive white Gaussian noise signals having zero means, they partially cancel out each other. Consequently, the SNR in f(t) may increase due to diversity.

More generally, a diversity system may combine *n* signals $f_{i}\left(t\right)$ that have, due to the uncorrelated channel gains, different signal amplitudes *a* as described by Equation (1) [17]:

(1) $$f\left(t\right)=\sum_{i=1}^{n}a_{i}f_{i}\left(t\right).$$

Combining the outputs of several antennas can increase the detected signal strength at a receiver [18] to improve communication robustness and reliability. There are existing several diversity techniques that can be utilized.

The example above describes time diversity as the two signals $f_{1}\left(t\right)$ and $f_{2}\left(t\right)$ reach the detector at different times. Separating receiver antennas in space achieves independent signal paths between sender and receiver. This technique is called space diversity. In frequency diversity systems, signals experience uncorrelated fading resulting from separate transmit frequencies. Furthermore, in polarization diversity, signals are received either along the horizontal or the vertical polarization. Polarization diversity has the advantage that it requires no spatial separation of the antennas [16].

Combining multiple versions of the same signal $f_{i}\left(t\right)$ according to Equation (1) is common to the above-mentioned diversity methods. However, if the multiple versions $f_{i}\left(t\right)$ are not in phase during combination, the resulting signal amplitude could even be lower than the originally transmitted signal amplitudes due to destructive interference. Diversity systems usually take care about this by using some sort of a phase-control technique [17].

**Figure 1:**
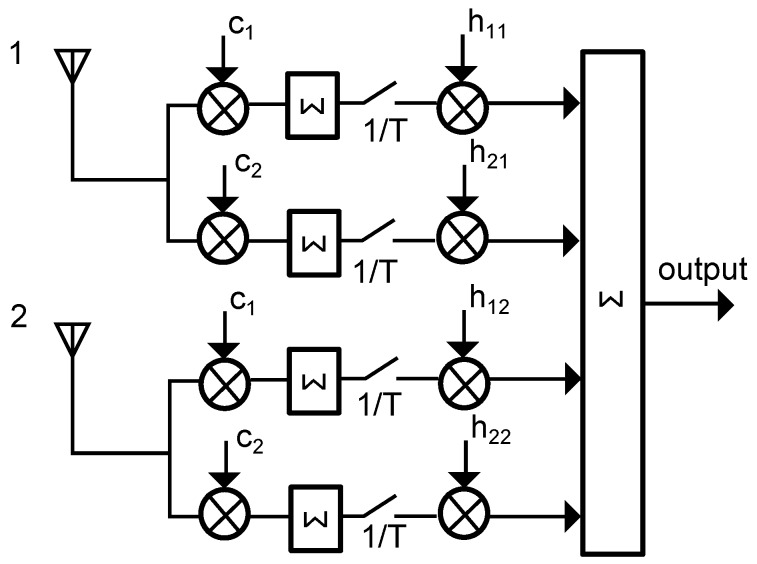
Schematic of a receiver with maximal ratio combining diversity for two input channels.

Phase alignment can be achieved, for example, by a cophase and sum circuit that can be realized, for instance, as a rake receiver that has the ability to demodulate each incoming signal independently. By correlating the incoming signals to delayed replica versions, a rake receiver can detect and reduce the relative phase offsets originating from the multipath propagation [19]. Figure 1 illustrates a generic rake receiver with two input channels [19]. The incoming signal of each antenna is processed in two channels to correlate the I- and Q-codes depicted as $c_{i}$ in Figure 1, where *i* resembles the I- and Q-parts of the incoming signals. After correlation, the signals are sampled at frequency 1/T, depicted by the switch in Figure 1. Then, each signal is amplified by the corresponding gain factor $h_{ij}$, where *j* resembles the antenna path. Finally, the output is achieved by summing all processed signals [19]. Resulting from its rather complex structure, a rake receiver usually has energy requirements in the mW area.

There exist mainly four diversity systems that can be combined with diversity techniques. Brennan [17] categorized them into scanning diversity, selection diversity, maximal-ratio combining, and equal-gain diversity. The latter two methods combine several independent sinusoidal signals to increase the signal strength. Their phases need to be aligned to avoid destructive interference before summation, in order to achieve this. For reasons of simplicity, we quickly review the concepts of selection diversity and equal-gain diversity in the following two paragraphs. More details about the methods as well as the other mentioned diversity techniques can be found in literature—for example, in [14,17].

### 2.1. Selection Diversity

**Figure 2:**
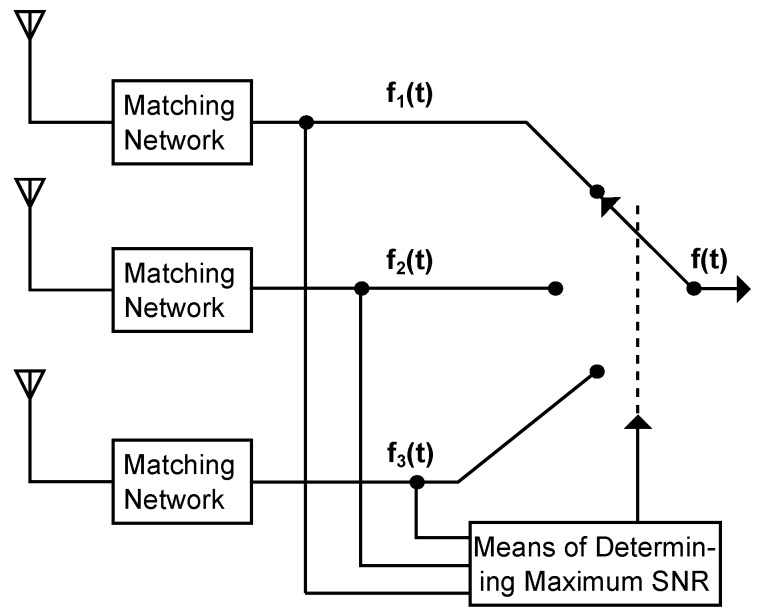
Schematic of a selection diversity system where the receiver is always connected to the antenna with the highest RSSI value.

For any incoming signal, a selection diversity system connects the receiver always to the antenna that has the highest RSSI (received signal strength indicator) value. All other signals do not contribute to f(t), as illustrated in Figure 2 [17], which sketches a selection diversity system consisting of three antennas that receive fading signals. The antenna with the highest RSSI value can be determined, for example, by comparing the short term averages of the incoming signals on all antennas. This antenna is then connected to the receiver. The average RSSI value detected at a receiver clearly increases compared to that of a single branch system. However, selection diversity is not an optimal diversity system, as it does not use all present diversity branches simultaneously [14].

### 2.2. Equal-Gain Diversity

In an equal-gain diversity system, the signals from the diversity branches are first co-phased and then summed at equal gain. An equal-gain diversity system provides a performance that is only marginally inferior to maximal-ratio combining systems and superior to selection diversity [14].

### 2.3. Discussion on Diversity Systems

Equal-gain systems can successfully increase the signal strength at their output only if the noise signals are uncorrelated and the data signals are correlated, as already pointed out by [20]. If there is a highly correlated noise signal present at the antennas, equal-gain-combining systems degrade in their performance and selection diversity can achieve a higher signal-to-noise ratio of the output signals then the other techniques. In addition, for practical reasons, selection diversity systems often measure the received signal strength indicator and not the signal-to-noise ratio, which also degrades their efficiency in high noise areas or in the case of co-channel interferences. To mitigate this effect, some selection diversity systems additionally measure the present bit error rate and if it falls below a certain threshold, they switch to another antenna, and it also has a lower received signal strength indicator [21].

## 3. Hardware

The diversity node presented here uses the low-frequency wake-up receiver AS3932. This device supports the build up of a selection diversity system as introduced in Section 2.1, as it features three input channels. If several antennas are connected to the receiver, the AS3932 always selects the channel that has the highest RSSI value [22]. The node introduced in this section uses the AS3932 low-frequency wake-up receiver to implement an equal gain diversity system to further improve the wake-up reliability as discussed in Section 2.2.

**Figure 3:**
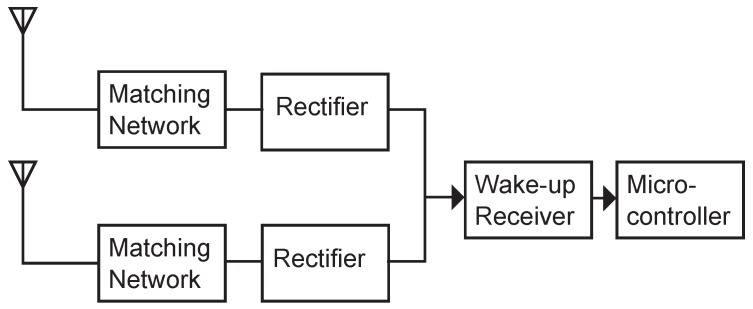
Block diagram of a low-power wake-up receiver with antenna diversity. Each diversity branch consists of antenna, matching network and rectifier.

Figure 3 illustrates the block diagram of the proposed diversity system. As can be seen, the two antenna paths both include a matching network and a rectifier to demodulate the incoming wake-up messages. The two branches are combined after the demodulation stage and then fed into the wake-up receiver.

**Figure 4:**
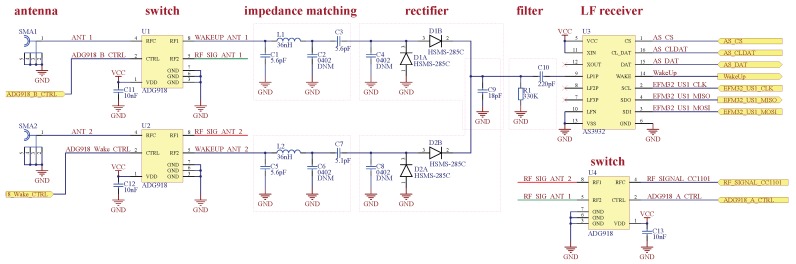
Schematic of the receiver front-end with two antennas including matching networks rectifiers AS3932 wake-up receiver and antenna switches.

Figure 4 depicts the schematic of the diversity system in further detail including the antenna switches (ADG918), the matching networks and the rectification stage of each antenna path. The two identical rectifiers are voltage doublers consisting of HSMS-285C zero bias Schottky diodes that are designed for input powers below −20 dBm and have a typical sensitivity of −57 dBm at 915 MHz [23]. Both branches are then combined to achieve constructive interference.

**Figure 5:**
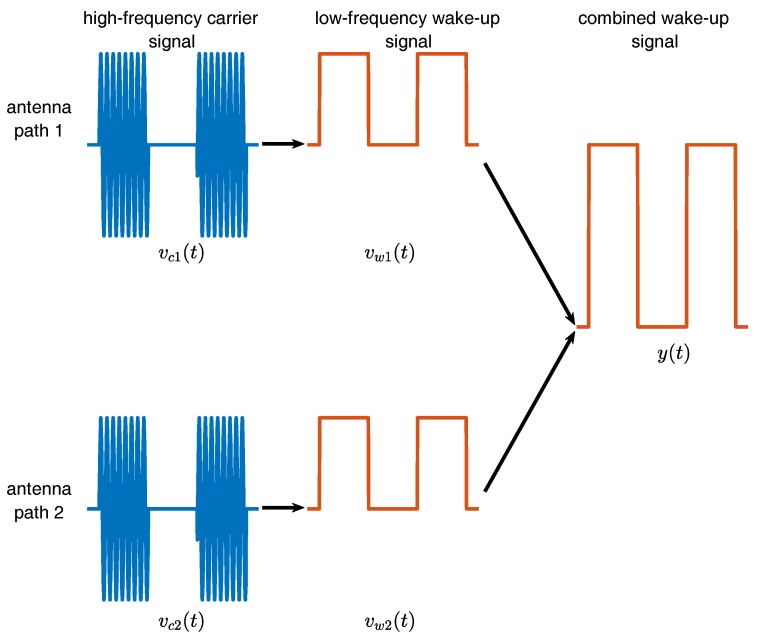
Simplified illustration of the signal chain starting with the on-off-keying modulated high-frequency carrier signals received by the two antennas, the low-frequency wake-up signals after demodulation and the combined wake-up signal.

Figure 5 illustrates simplified two wake-up signals received at the two antennas. It is assumed that both antennas receive a signal of the same amplitude for reasons of simplicity. The high-frequency carrier signals $v_{c1}\left(t\right)$ and $v_{c2}\left(t\right)$ as illustrated in blue in Figure 5 are on-off-keying modulated to carry the low-frequency wake-up message. The demodulation stage filters the high-frequency carrier signal to achieve the low-frequency wake-up signals $v_{w1}\left(t\right)$ and $v_{w2}\left(t\right)$. Finally, the two low-frequency signals are added to form the combined wake-up signal y(t) according to Equation (2):

(2) $$y\left(t\right)=v_{w1}\left(t\right)+v_{w2}\left(t\right)=a_{1}\cos\left(\omega_{0}t+\phi_{1}\right)+a_{2}\cos\left(\omega_{0}t+\phi_{2}\right),$$

with $\omega_{0}$ being the wake-up frequency, for example 125 kHz, and $\phi_{i}$ being a possible phase shift of the two wake-up signals. Obviously, Equation (2) resembles Equation (1) with y(t) being the combination of the two antenna branches.

It is obvious that destructive interference of two sinusoidal signals of the form $y_{i}\left(t\right)=A_{i}e^{j(\omega +\phi_{i})t}$ with i=1,2 happens when the phase shift ($\phi_{2}-\phi_{1}$) is in the range $2/3\pi <\phi_{2}-\phi_{1}<4/3\pi$. The down converted signal at the wake-up receiver input is at 125 kHz. In case of destructive interference and by using the relation Δt=Δφ/ω, a time offset Δt in the range of 2.66 × 10^−6^ s <Δt < 5.33 × 10^−6^ s can be calculated. As the signals are transmitted at speed of light c= 300 × 10^6^ $ms^{-1}$, this translates to an antenna separation between 800 m and 1600 m, which is impossible with the hardware used in this work.

As can be seen, the block diagram in Figure 3 resembles an equal gain system as introduced in Section 2.2. Furthermore, the utilized diversity scheme is polarization diversity, as it requires less space than, for example, a space diversity system.

**Figure 6:**
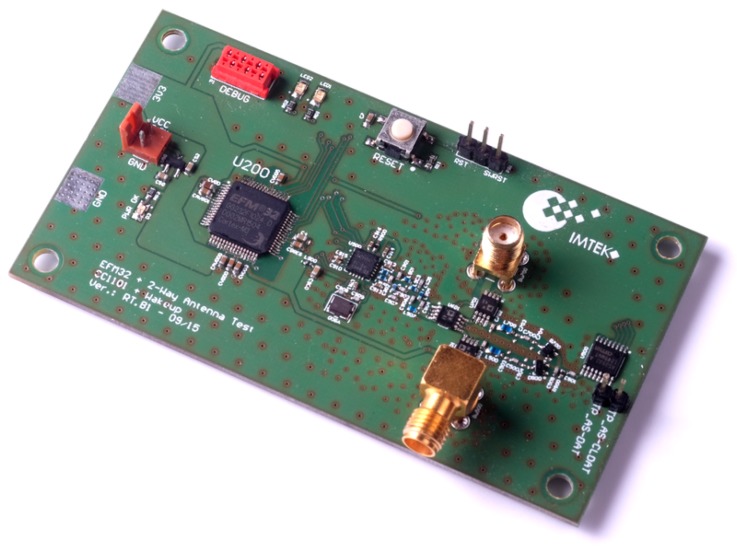
Photo of the wireless sensor node with equal gain diversity.

Figure 6 shows a photo of the wireless sensor node with antenna diversity. To feature multiple antennas, the board is equipped with two antenna ports, which are connected to an ADG918 antenna switch from Analog Devices. By inserting a third antenna switch, it is possible to use both antennas as input and output. Due to the three extra antenna switches, the node has an additional power consumption of less than 3 µA compared to the power consumption of the node introduced above.

**Figure 7:**
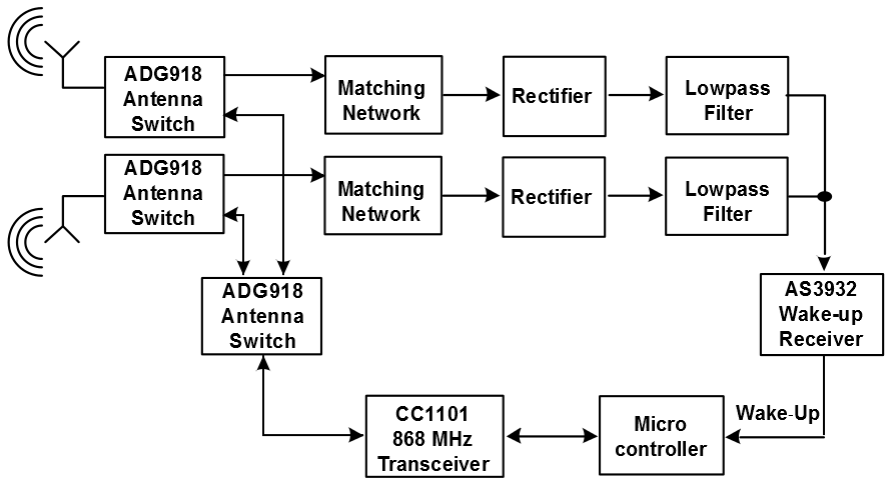
Schematic of the diversity system.

Of course, the additional components introduce some extra costs. However, the benefit of combining the low-frequency signals over combining the high-frequency signals using a classical equal-gain diversity technique that uses, for example, a Wilkinson splitter, clearly is the not required phase-control circuit as visualized in Figure 1. As shown above, destructive interference of the low-frequency signals is virtually not possible. Figure 7 illustrates the block diagram of the sensor node. As discussed above, the low frequency wake-up receiver AS3932 has a typical sensitivity of 100 µV RMS (root mean square).

## 4. Experimental Results

To verify the design and to test the performance of the wake-up diversity node, several experiments were performed. First, a static experiment was used to verify expected input signal gain of about 3 dB. Then, the performance of the wake-up diversity system was investigated in a multipath environment. Throughout the tests, the wake-up diversity node was equipped with two identical monopol antennas with omnidirectional radiation patterns and an antenna gain of maximal 2 dBi and 50 Ω impedance. The antennas were mounted at an angle of 90° to each other to reduce the correlation coefficient between the two antennas and to achieve a polarization diversity system.

### 4.1. Antenna Port Impedances

**Figure 8:**
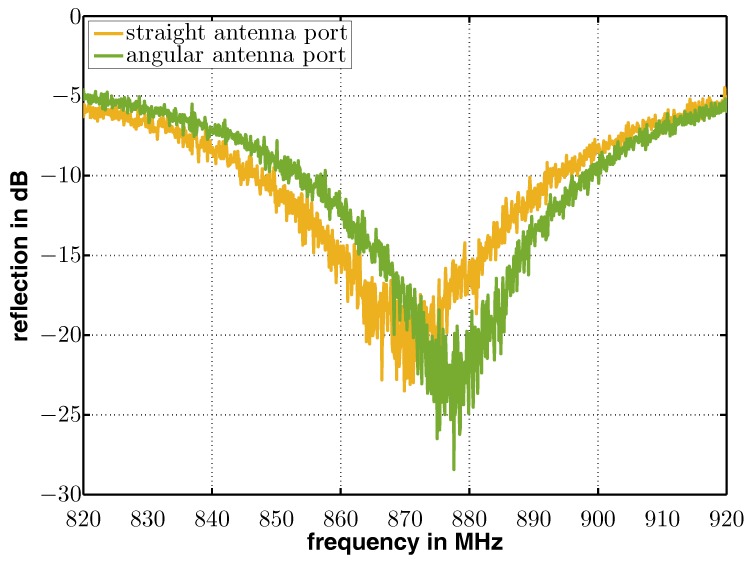
Reflections measured at the straight (yellow) and angular (green) antenna input ports over frequency.

To verify the impedances of both antennas, a frequency sweep from 820 Mhz to 920 Mhz was performed and the reflections were measured with a network analyzer. The measurement results are plotted in Figure 8 that illustrates the reflection of the straight antenna in yellow and the reflection of the angular antenna in green. As can be seen, the minimum reflection is at approximately 870 MHz for the straight antenna and at approximately 878 MHz in the case of the angular antenna. With a reflection below −15 dB, both antennas perform very well in the frequency region of interest that is around 870 MHz.

### 4.2. Static Measurements

**Figure 9:**
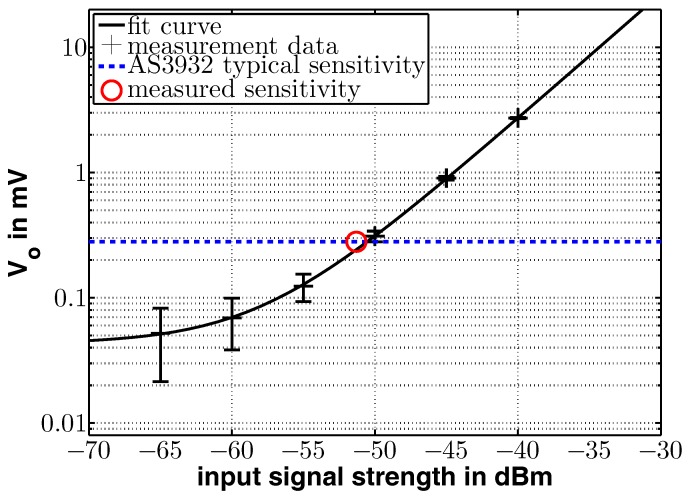
Measured voltage at the rectifier output with one active antenna. The typical sensitivity of the AS3932 is depicted as the dotted line and the red circle shows the measured sensitivity.

At first, a signal generator was connected to the antenna ports of the board, feeding them consecutively with a 868 MHz signal at different input levels. Since both measurements achieved almost equal results, Figure 9 shows the output voltage over the input level for the straight antenna, only. The measurements with the angular antenna revealed similar results and can be found in the third column of Table 1.

An exponential function was used to fit the measurement data, superimposed on a constant noise level, that is $y_{1}=a+b\exp\left(cx\right)$ with a=0.043, b=26,734 and c=0.23. The theoretical sensitivity of the AS3932 intersects the fitted curve at −50.6 dBm. The circle in Figure 9 shows the sensitivity measured by using the AS3932 receiver: when the receiver did not further react to the input signals, its sensitivity limit was reached. Using one antenna input feed, it was found at −51.3 dBm, which fits very well to the results reported by [6] and as discussed above.

**Figure 10:**
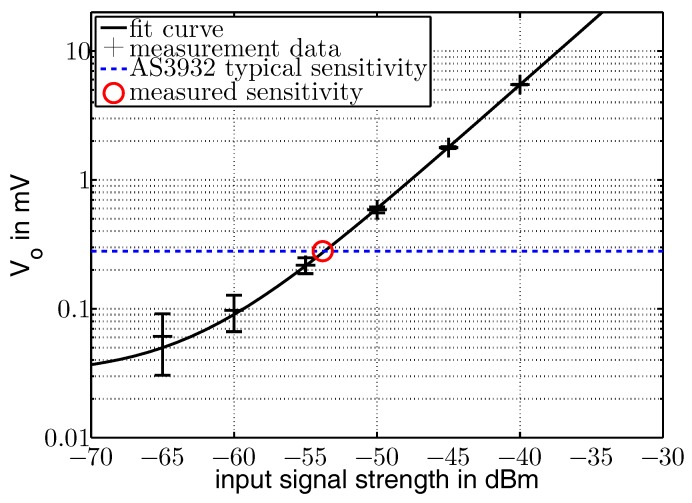
Measured voltage at the rectifier output with two active antennas. Typical sensitivity of the AS3932 is depicted as the blue dotted line. The red circle marks the measured sensitivity.

In a second step, a second signal generator was connected to the other antenna input port, also feeding it with a 868 MHz signal. Figure 10 shows the data curve fitted with an exponential function of the form $y_{2}=a+b\exp\left(cx\right)$ with a=0.031, b=45,410 and c=0.226. The intersection of the fitted curve and the theoretical sensitivity line is at −53.7 dBm.

The voltage where the AS3932 did no longer sense the input signal was found to be at −53.8 dBm, depicted as a circle in Figure 10. This is a gain of around 3 dB compared to the system with one antenna. Table 1 lists the experimentally received data that is also plotted in Figure 9 and Figure 10 plus the measured voltage for the case of only the angular antenna input powered.

**Figure 11:**
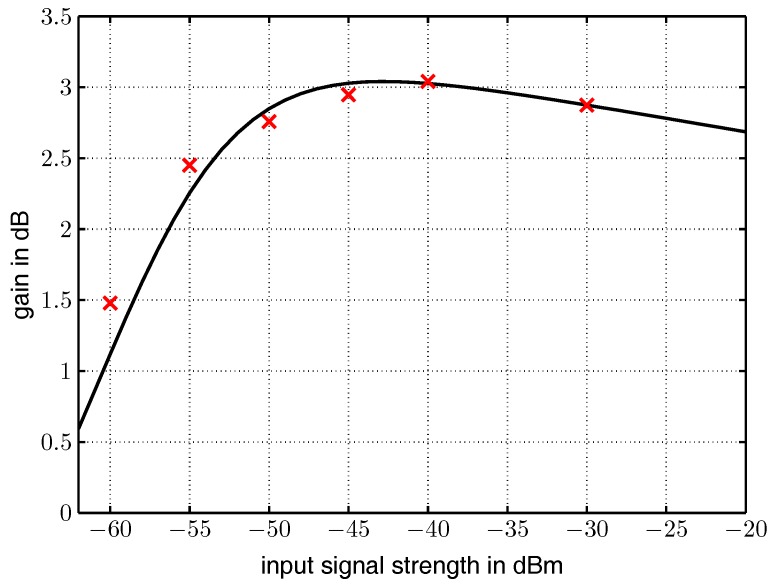
Experimentally measured gain in dB of the two antenna diversity system.

Table 2 summarizes the experimentally identified wake-up sensitivities for the four possible combinations of the experiments above. Figure 11 compares the input signal strength with one receiving antenna to the input signal strength with two receiving antennas in dB. The red crosses illustrate the measurement data the solid curve depicts the fitted curve achieved by dividing $y_{1}$ by $y_{2}$. It can be observed that the signal gain reaches approximately 3 dB in the area where the input signal strength varies from − 50 dBm to −30 dBm, which correlates well to Equation (2) with $a_{1}=1$ and $a_{2}=1$. Below an input signal strength of −50 dBm and above −30 dBm, the gain decreases, probably because of decreased efficiency of the rectifier diodes that are specified for the region from −20 dBm to −57 dBm [23].

### 4.3. Measurements in Multipath Environment

**Figure 12:**
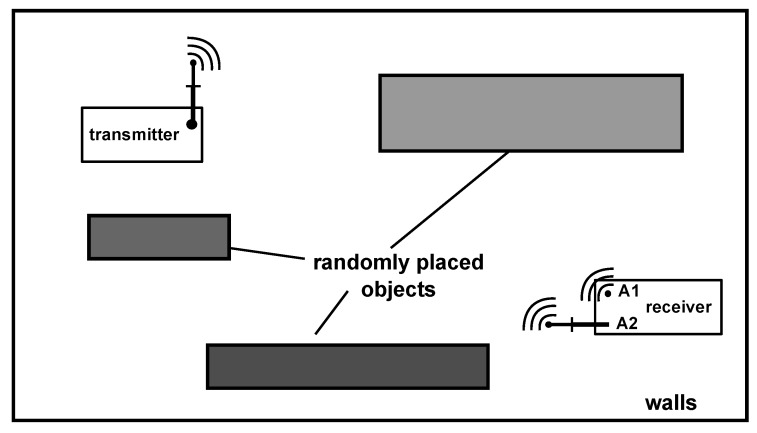
Multipath laboratory setup with randomly placed objects.

For the second test, the diversity node was equipped with antennas and the 868 MHz wake-up message was transmitted wirelessly, but generated by a signal generator to be able to accurately control the output power. Figure 12 depicts the test setup. The sender was placed at a fixed position in the laboratory, and the receiver was positioned at several different locations. Inside the laboratory were several randomly placed objects like chairs, tables, shelfs and general laboratory equipment. In summary, the objects generated an multipath environment suitable to test the diversity wake-up system.

Table 3 reports the measurement results at the six positions. The first column gives the position, the second column gives the required transmit power to wake-up the node with one antenna connected in straight position. The third column is the required transmit power to wake-up the node with one antenna connected in angular position and the last column was the required transmit powers when both antennas are connected to the node.

**Figure 13:**
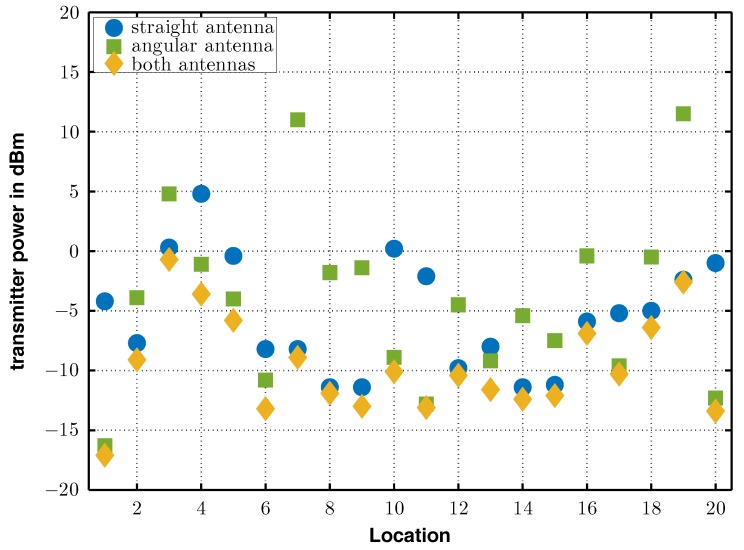
Required power to wake-up the receiver at different positions.

Assume that a selection diversity system chooses the signal with the highest RSSI value. In this particular experiment, the equal-gain diversity system presented here outperformed both single antenna solutions about approximately 0.2 dB to 2.5 dB and consequently successfully demonstrated its superior performance compared to selection diversity. Figure 13 illustrates the required transmitter powers at the 20 locations graphically. The blue circles show the results for the straight antenna system, the green squares for the angular antenna system and the yellow markers depict the equal-gain diversity results. In this particular experiment, the equal-gain system outperformed both of the other configurations in all measurements, as can be seen in Figure 12.

**Figure 14:**
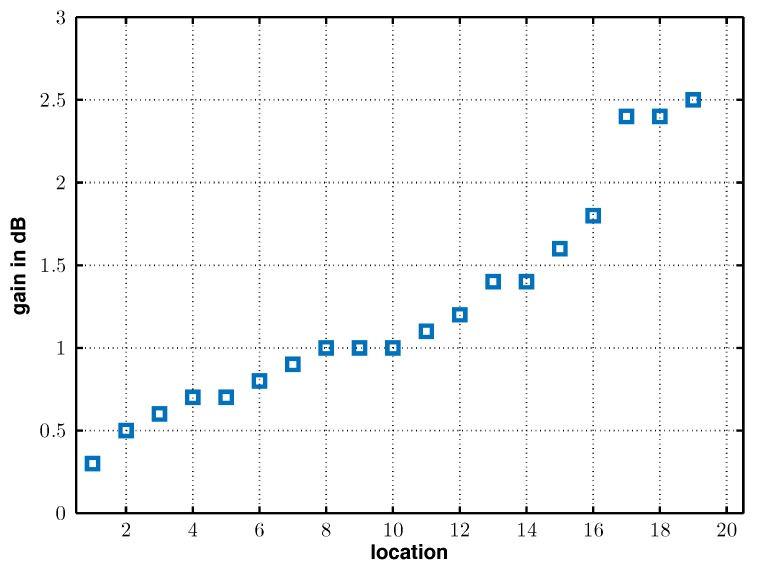
Gain achieved by the equal-gain diversity wake-up receiver system over the selection diversity wake-up system for the 20 locations in ascending order.

To visualize the benefit of the equal-gain diversity wake-up system compared to a selection diversity wake-up system, Figure 14 plots the signal strengths differences required to wake-up the wireless node using the equal-gain diversity method compared to selection diversity in ascending order for the 20 observed locations. It can be seen that the gain of the equal-gain diversity system lies between 0.2 dB and 2.5 dB. Adding signals received by both antennas in an equal manner is also a possible drawback of the equal-gain diversity system. For example, if one antenna receives a strong noise signal, the effective signal-to-noise-ratio at the receiver input will be reduced compared to the signal strength that would be received with only one receiving antenna that has a high signal-to-noise ratio. On the other hand, most selection diversity systems choose the channel with the apparent highest received input signal strength, but not the channel with the highest signal-to-noise-ratio.

## 5. Conclusions

The ultra low-power wireless sensor node presented in this article successfully demonstrated the use of equal-gain-combining in combination with a low-frequency wake-up receiver. Resulting from the wake-up frequencies in the kilohertz range, energy-demanding phase control circuits are not required. The implemented and investigated equal-gain wake-up diversity node requires only two additional antenna switches to combine the multiple input signals and that increase the power consumption only marginally. The polarization diversity technique was implemented, although this is no limitation and other diversity techniques like space diversity could also be utilized. It could be verified that the antenna diversity achieved 3 dB gain under ideal laboratory conditions where the signals were free from noise, but not phase aligned. A laboratory multipath environment experiment confirmed the performance gain between 0.2 and 2.5 dB of the equal-gain wake-up diversity system.

## Figures and Tables

**Table 1:** Output voltage of rectifier at decreasing input signal levels using different antenna configurations.

Input Level (dBm)	Measurement (mV)		
	Straight Antenna	Angular Antenna	Both Antennas
−10	1050	1036	1190
−20	234	228	316
−30	26.9	26.1	52.1
−40	2.76	2.69	5.49
−45	0.90	0.90	1.78
−50	0.31	0.31	0.59
−55	0.12	0.14	0.22
−60	0.07	0.08	0.10
−65	0.05	0.06	0.06

**Table 2:** The table shows the wake-up sensitivities of the wireless sensor node achieved by measuring the rectified voltage and by feeding the AS3932 with a 868 MHz input signal.

	One Antenna	Two Antennas
voltage measurement	−50.6 dBm	−53.7 dBm
wake-up signal	−51.3 dBm	−53.8 dBm

**Table 3:** Required sender power to wake-up the receiver measured at 20 different multipath locations and different antenna configurations.

Location	Straight Antenna (dBm)	Angular Antenna (dBm)	Both Antennas (dBm)
1	−4.2	−16.3	−17.1
2	−7.7	−3.9	−9.1
3	+0.3	+4.8	−0.7
4	+4.8	−1.1	−3.6
5	−0.4	−4.0	−5.8
6	−8.2	−10.8	−13.2
7	−8.2	+11.0	−8.9
8	−11.4	−1.8	−11.9
9	−11.4	−1.4	−13.0
10	+0.2	−8.9	−10.1
11	−2.1	−12.8	−13.1
12	−9.8	−4.5	−10.4
13	−8.0	−9.2	−11.6
14	−11.4	−5.4	−12.4
15	−11.2	−7.5	−12.1
16	−5.9	−0.4	−6.9
17	−5.2	−9.6	−10.3
18	−5.0	−0.5	−6.4
19	−2.4	+11.5	−2.6
20	−1.0	−12.3	−13.4

## References

[1] Puccinelli D., Haenggi M. (2005). Wireless sensor networks: Applications and challenges of ubiquitous sensing. Circuits Syst. Mag. IEEE.

[2] Hoflinger F., Gamm G.U., Albesa J., Reindl L.M. Smartphone remote control for home automation applications based on acoustic wake-up receivers. Proceedings of the 2014 IEEE International Instrumentation and Measurement Technology Conference (I2MTC).

[3] Kumberg T., Tannhaeuser R., Schink M., Schneid S., Koenig S., Schindelhauer C., Reindl L.M. Wireless Wake-up Sensor Network for Structural Health Monitoring of large-scale Highway Bridges. Proceedings of the International Conference on Performance-based and Life-cycle Structural Engineering.

[4] Kumberg T., Kokert J., Younesi V., Koenig S., Reindl L.M. Wake-up transceivers for structural health monitoring of bridges. Proceedings of the SPIE Smart Structures and Materials Nondestructive Evaluation and Health Monitoring.

[5] Kumberg T., Schneid S., Reindl L.M. (2017). A wireless sensor network using GNSS receivers for a short-term assessment of the modal properties of the neckartal bridge. Appl. Sci..

[6] Gamm G.U., Kostic M., Sippel M., Reindl L.M. (2012). Low-power sensor node with addressable wake-up on-demand capability. Int. J. Sens. Netw..

[7] Piyare R., Murphy A.L., Kiraly C., Tosato P., Brunelli D. (2017). Ultra Low Power Wake-Up Radios: A Hardware and Networking Survey. IEEE Commun. Surv. Tutor..

[8] Kumberg T., Tannhaeuser R., Gamm G.U., Reindl L.M. Energy improved wake-up strategy for wireless sensor networks. Proceedings of the Sensors and Measuring Systems 2014; 17. ITG/GMA Symposium.

[9] Kumberg T., Schink M., Reindl L., Schindelhauer C. (2017). T-ROME: A Simple and Energy Efficient Tree Routing Protocol for Low-Power Wake-up Receivers. Ad Hoc Netw..

[10] Kumberg T., Reindl L., Moharrami M., Schindelhauer C. Improving the performance of the cross-layer wake-up routing protocol T-ROME. Proceedings of the 13th International Wireless Communications and Mobile Computing Conference (IWCMC).

[11] Kumberg T., Schindelhauer C., Reindl L. (2017). Exploiting Concurrent Wake-Up Transmissions Using Beat Frequencies. Sensors.

[12] Kumberg T., Tannhaeuser R., Reindl L.M. Using antenna diversity to improve wake-up range and probability. Proceedings of the Progress In Electromagnetics Research Symposium.

[13] Karl H., Willig A. (2007). Protocols and Architectures for Wireless Sensor Networks.

[14] Rappaport T.S. (1996). Wireless Communications: Principles and Practice.

[15] Seybold J.S. (2005). Introduction to RF Propagation.

[16] Murch R.D., Letaief K.B. (2002). Antenna systems for broadband wireless access. IEEE Commun. Mag..

[17] Brennan D. (2003). Linear diversity combining techniques. Proc. IEEE.

[18] Dietrich C.B., Dietze K., Nealy J.R., Stutzman W.L. (2001). Spatial, polarization, and pattern diversity for wireless handheld terminals. IEEE Trans. Antennas Propag..

[19] Wallace M., Walton J. (2001). Method and Apparatus for Antenna Diversity in A Wireless Communication System. U.S. Patent.

[20] Parsons J., Henze M., Ratliff P., Withers M.J. (1975). Diversity techniques for mobile radio reception. Radio Electron. Eng..

[21] Todd S.R. (1999). Diversity Antenna Selection. US Patent.

[22] AS3932—Programmable 3D Low Power LF Wake-up Receiver IC—AMS. http://ams.com/eng/Products/Wireless-Connectivity/Wireless-Sensor-Connectivity/AS3932.

[23] HSMS-285C-Broadcom Limited. https://www.broadcom.com/products/wireless/diodes/schottky/hsms-285c.

